# Whole-genome sequencing reveals transmission of vancomycin-resistant *Enterococcus faecium* in a healthcare network

**DOI:** 10.1186/s13073-015-0259-7

**Published:** 2016-01-12

**Authors:** Hayley J. Brodrick, Kathy E. Raven, Ewan M. Harrison, Beth Blane, Sandra Reuter, M. Estée Török, Julian Parkhill, Sharon J. Peacock

**Affiliations:** Department of Medicine, University of Cambridge, Addenbrooke’s Hospital, Box 157, Hills Road, Cambridge, CB2 0QQ UK; Cambridge University Hospitals NHS Foundation Trust, Hills Road, Cambridge, CB2 0QQ UK; Cambridge Public Health England Microbiology and Public Health Laboratory, Box 157, Hills Road, Cambridge, CB2 0QQ UK; Wellcome Trust Sanger Institute, Wellcome Genome Campus, Hinxton, Cambridge, CB10 1SA UK; London School of Hygiene and Tropical Medicine, London, WC1E 7HT UK

## Abstract

**Background:**

Bacterial whole-genome sequencing (WGS) has the potential to identify reservoirs of multidrug-resistant organisms and transmission of these pathogens across healthcare networks. We used WGS to define transmission of vancomycin-resistant enterococci (VRE) within a long-term care facility (LTCF), and between this and an acute hospital in the United Kingdom (UK).

**Methods:**

A longitudinal prospective observational study of faecal VRE carriage was conducted in a LTCF in Cambridge, UK. Stool samples were collected at recruitment, and then repeatedly until the end of the study period, discharge or death. Selective culture media were used to isolate VRE, which were subsequently sequenced and analysed. We also analysed the genomes of 45 *Enterococcus faecium* bloodstream isolates collected at Cambridge University Hospitals NHS Foundation Trust (CUH).

**Results:**

Forty-five residents were recruited during a 6-month period in 2014, and 693 stools were collected at a frequency of at least 1 week apart. Fifty-one stool samples from 3/45 participants (7 %) were positive for vancomycin-resistant *E. faecium*. Two residents carried multiple VRE lineages, and one carried a single VRE lineage. Genome analyses based on single nucleotide polymorphisms (SNPs) in the core genome indicated that VRE carried by each of the three residents were unrelated. Participants had extensive contact with the local healthcare network. We found that VRE genomes from LTCF residents and hospital-associated bloodstream infection were interspersed throughout the phylogenetic tree, with several instances of closely related VRE strains from the two settings.

**Conclusions:**

A proportion of LTCF residents are long-term carriers of VRE. Evidence for genetic relatedness between these and VRE associated with bloodstream infection in a nearby acute NHS Trust indicate a shared bacterial population.

**Electronic supplementary material:**

The online version of this article (doi:10.1186/s13073-015-0259-7) contains supplementary material, which is available to authorized users.

## Background

Over the last decade the introduction of a series of infection control initiatives in acute healthcare environments in the UK has been associated with a dramatic decline in nosocomial infections caused by methicillin-resistant *Staphylococcus aureus* (MRSA) and *Clostridium difficile* [1, 2]. By contrast, the prevalence of bacteraemia caused by vancomycin-resistant enterococci (VRE) has been more resistant to change. Voluntary surveillance in England, Wales and Northern Ireland shows that bacteraemia caused by vancomycin-resistant *Enterococcus faecium* rose from 16 % to 24 % between 2010 and 2014 [3], with a similar increase observed across Europe [1]. VRE infections are associated with increased morbidity, mortality, healthcare costs and duration of hospital stay compared with vancomycin-susceptible (VSE) infections [4, 5].

Accurate identification of VRE reservoirs and transmission routes provides strategic direction to infection control interventions. Individuals in long-term care facilities (LTCF) frequently move through healthcare networks and are potential reservoirs for VRE [6, 7]. Pathogen transmission between people in long-term residential and acute care facilities is likely to be common [8], but investigation is hampered by the low resolution of current typing methods, which cannot distinguish between isolates belonging to the same clone [9, 10]. Whole-genome sequencing (WGS) offers improved resolution, and has been used to delineate pathogen transmission of a range of bacterial species at local, national and global scales [11–14]. WGS also provides the sensitivity to detect genetic diversity of the same clone in an individual [15, 16]. Despite a growing body of data, microbial WGS has not been used to capture a dynamic picture of VRE carriage and transmission within and between interconnected healthcare facilities over time. The aim of this study was to define VRE carriage by residents of a LTCF over a 6-month period, and to use WGS to describe the genetic relatedness of isolates within and between residents, document whether VRE was transmitted in the LTCF over time, and compare these data with the genomes of bloodstream isolates in patients in the nearby acute hospital.

## Methods

### Study design, setting and participants

We conducted a prospective observational cohort study of faecal VRE carriage in residents of a LTCF over a 6-month period in Cambridge, United Kingdom (UK). The LTCF had 105 beds and was subdivided into five physically separated units, to which residents were assigned based on cognitive impairment and physical disability.

### Ethics, consent and permissions

All residents admitted to the LTCF during the study period were eligible for inclusion. Residents were excluded if they refused consent, were on an end-of-life care pathway, or were strongly resistant to basic personal care. Written informed consent was obtained from study participants prior to enrolment. For participants who lacked mental capacity, we obtained assent from their consultee. The study protocol was approved by the National Research Ethics Service (REC ref: 13/LO/1278) and the Cambridge University Hospitals NHS Foundation Trust Research and Development department (ref: A093007). All study procedures were performed in accordance with the Declaration of Helsinki.

### Study procedures

Data were collected from medical records and nursing care plans. This included demographics (age, gender, unit of residence), presence of co-morbidities (Charlson co-morbidity Index), presence of urinary or faecal incontinence or a urinary catheter, a history of VRE carriage or infection in the 12 months prior to enrolment, and infection and antibiotic use during the study. Data were also collected on healthcare contact (inpatient admission, outpatient clinic attendance, or general practitioner (GP) surgery visit) in the 12 months prior to enrolment and during the study period. Stool samples were collected at recruitment and then repeatedly (up to once a week) until the end of the study period, discharge from the LTCF or death.

### Laboratory procedures

Stool samples were cultured within 24 h of collection on Monday to Friday, or within 48 h if collected at weekends. A 10 μL loopful was added to 5 mL Brain-Heart Infusion Broth (BHI) (Merck, Darmstadt, Germany) containing 3 μg/mL vancomycin (Sigma-Aldrich, St Louis, MO, US) and incubated at 37 °C in air at 100 rpm for 24 h. A total of 200 μL was plated onto Brilliance VRE agar (Oxoid, Basingstoke, UK) and incubated at 37 °C for 48 h. Purple or blue colonies (putative *E. faecium* or *E. faecalis*, respectively) were sub-cultured on Columbia Blood agar (CBA, Oxoid, Basingstoke, UK), incubated at 37 °C for 48 h, and the species confirmed using matrix-assisted laser desorption/ionization time-of-flight mass spectrometry (MALDI-TOF) (Bruker Daltoniks, Bremen, Germany). Antimicrobial susceptibility was determined using the Vitek2 instrument (BioMérieux, Marcy l’Etoile, France) and the AST-P607 card. All stools positive for VRE were cultured for VSE. A 10 μL loopful was plated onto Brilliance UTI agar (Oxoid, Basingstoke, UK). Presumptive enterococci were sub-cultured on CBA containing a 5 μg vancomycin disc (Oxoid, Basingstoke, UK). Colonies growing at the edge of the zone of inhibition were selected for identification and antimicrobial susceptibility testing as above.

### Bacterial sequencing and analysis

Genomic DNA was extracted from a pure bacterial culture based on a single colony using the QIAxtractor (QIAgen, Hilden, Germany). Library preparation was based on the Illumina protocol, and sequencing was performed on an Illumina HiSeq2000 with 100-cycle paired-end runs. Sequence reads were assembled using Velvet and mapped to the *E. faecium* reference genome Aus0004 (European Nucleotide Archive (ENA) accession number CP003351) using SMALT [17]. Mobile genetic elements (MGEs) were identified (genes annotated as plasmid-, phage-, IS- or transposon-associated) and removed. Gubbins was used to remove regions of recombination [18], creating a core genome. Core genome single nucleotide polymorphisms (SNPs) were used to estimate maximum likelihood trees using RAxML [19] with 100 bootstraps and a midpoint root. The multilocus sequence type (ST) was derived from sequence data using an in-house script and the MLST database [20]. *vanA* and *vanB* were detected by *in silico* polymerase chain reaction (PCR) using published primers [21, 22]. Isolates positive for *vanA* were mapped to a Tn*1546* reference (ENA accession number M97297). The nucleotide sequences of the *Tn1546* transposon, and those up- and downstream of the transposon, in each isolate were identified as described by Howden *et al.* [23] using Artemis [24], and queried against each other and nucleotide databases using BLAST. Isolates positive for *vanB* were mapped to Aus0004 Tn*1549* [25] and up- and downstream nucleotide sequences identified as for *vanA.* Plasmid replicon (*rep)* typing was performed using *in silico* PCR and published primers [26, 27].

Whole-genome sequences were available for 45 *E. faecium* bloodstream isolates collected between January and December 2012 at the Cambridge University Hospitals NHS Foundation Trust (CUH) (S. Peacock, personal communication). These consisted of the first stored isolate for 45 patients, identified using the diagnostic microbiology laboratory database at CUH. Information on date of isolation and patient location was available. These isolates had been sequenced as part of a previous study, approved by the National Research Ethics Service (REC ref: 12/EE/0439) and the Cambridge University Hospitals NHS Foundation Trust R&D Department (ref: A092685). Sequence data for all study isolates have been submitted to the European Nucleotide Archive with the accession numbers shown in Additional file 1.

## Results

### Baseline characteristics

Forty-five of the 90 (50 %) eligible residents were recruited. Participants had a median age of 82 years (range, 40–104 years; interquartile range (IQR), 71–87 years), and 29 (64 %) were female. Three participants were lost to follow-up during the study because of death (n = 2) or transfer to another facility (n = 1). The median duration of residence in the study facility at the time of recruitment was 16 months (range, 5 days to 54 months; IQR, 6–41 months). The median Charlson co-morbidity Index was 6 (range, 0–10). Thirty-three (73 %) participants were incontinent of urine and faeces (three of whom had an indwelling urinary catheter), and two (5 %) participants had urinary incontinence alone. One participant (P7) had a history of VRE infection (of the urinary tract) in the 12 months prior to recruitment.

The majority of residents (38/45, 85 %) had previously resided in another healthcare facility. Movement of participants through the healthcare network is summarised in Table 1. Healthcare contact was extensive, with more than half of participants (n = 26, 58 %) having at least one episode of healthcare contact (hospital admission, outpatient clinic attendance or visit to a GP surgery) in the 12 months prior to recruitment, and six participants having between 1 and 5 (total of 19) episodes of healthcare contact during the study. There were 61 infections recorded in 23 (51 %) participants (median, 2 infections per case; range, 1–5) during the 6-month study period, although none of these were associated with VRE. The most common site of infection was the urinary tract (n = 33, 54 %), followed by respiratory tract (n = 16) and skin and soft tissue infection (n = 8). The focus of infection in four cases was not specified. All participants with a urinary catheter had at least one urinary tract infection recorded during the study period. The 23 participants received 63 courses of antibiotics (median, 2 courses; range, 1–6).

**Table 1:** Summary of healthcare contact for 45 study participants

Healthcare contact	Frequency
Place of residence before admission to study facility (n, %)	
Home	7 (46.7 %)
Hospital	17 (37.8 %)
Residential care^a^	21 (15.6 %)
Participants with healthcare contact 12 months before recruitment (n, %)	26 (57.7 %)
Total number of episodes	60
Median (range) per participant	2 (1–4)
Inpatient admission^b^	23/60 (38.3 %)
Outpatient attendance^c^	35/60 (58.3 %)
General practice attendance^d^	2/60 (3.3 %)
Participants with healthcare contact during study (n, %)	6 (13.3 %)
Total number of episodes	19
Median (range) per participant	2 (1–5)
Inpatient admission^b^	8
Outpatient attendance^c^	11
General practice attendance^d^	0

^a^Residential care – any nursing or residential home

^b^Admission to hospital for at least an overnight stay

^c^Outpatient department visit or visit to emergency department not resulting in admission

^d^Visit to GP surgery

### Carriage of vancomycin-resistant *E. faecium* by study participants

**Fig. 1:**
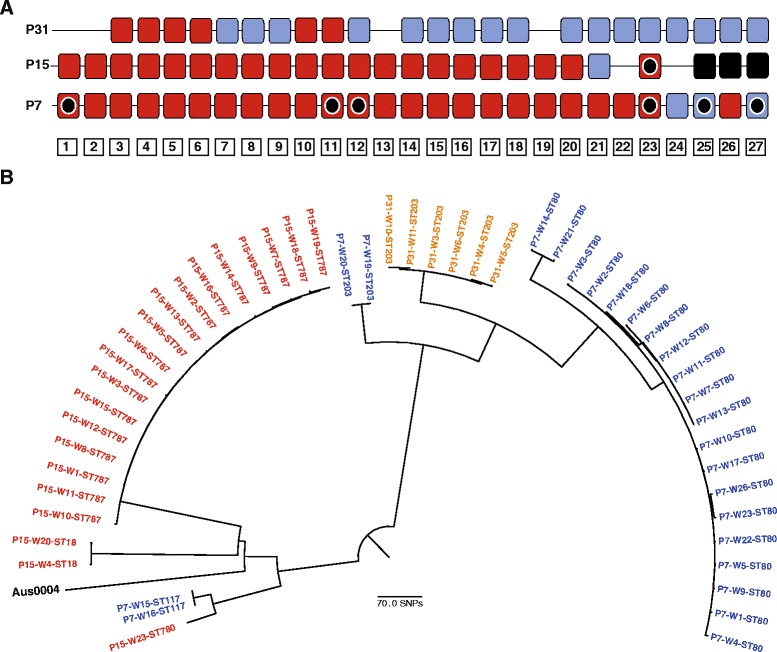
Epidemiological and genomic description of VRE isolated from stool of study participants*.* **a** Timeline of VRE positivity for three study participants, with week indicated at the bottom. Red, VRE isolated; blue, VRE not isolated; black, discharged; line, no sample available; black circle, admitted to local NHS Trust for at least 24 h. **b** Maximum likelihood tree based on core genome SNPs of 51 VRE isolates from three study participants. Each participant is identified by a unique colour, and labels show the participant number, week of isolation and ST

A total of 693 stools were collected over the 6-month study period, at a frequency of at least 1 week apart. Fifty-one stools from three participants (7 %) were positive for VRE, all of which were *E. faecium* (n = 24, 21 and 6 positive samples for P7, P15 and P31, respectively*)*. This included the participant (P7) who had a past history of VRE infection. Figure 1a shows the timing of positive and negative samples for each of the three cases. STs of the 51 VRE isolates were identified from the sequence data, which assigned the isolates to six STs (ST18, ST80, ST117, ST203, ST780 and ST787). A notable feature was that two residents (P7 and P15) each carried multiple STs with no overlap in STs between the two cases: P7 carried ST80 (isolated from 20 stool samples), ST117 (n = 2), and ST203 (n = 2), and P15 carried ST787 (isolated from 18 stool samples), ST18 (n = 2), and ST780 (n = 1). The third VRE positive resident (P31) carried a single ST that was assigned to ST203. *In silico* PCR demonstrated that the 51 VRE isolates carried *vanA.*

A phylogenetic tree for the 51 VRE isolates based on SNPs in the core genome is shown in Fig. 1b. P7 carried four genetically distinct lineages (two of which belonged to ST80), and P15 carried three, with no evidence for carriage of related strains by these two residents. The majority of VRE isolates from P7 and P15 each belonged to a single lineage (18/24 (75 %), and 18/21 (86 %)), with a median pairwise SNP difference within each of these two clusters of 3 (range, 0–6) and 1 (range, 0–6), respectively. P7 and P31 both carried VRE isolates belonging to ST203, but these were genetically distinct (median SNP difference of 74; range, 73–74), excluding transmission between them (Fig. 1b). The VRE-positive participants resided in different units. All three VRE positive participants had healthcare contact in the 12 months prior to the study, including hospital admission (5 episodes), outpatient clinic attendance (3 episodes) or attendance at Accident and Emergency Department without admission (1 episode), and one VRE positive participant (P7) had healthcare contact during the study consisting of four inpatient admissions and five outpatient clinic attendances.

To identify the genomic location of the *vanA* transposon and its relatedness within and between participants, we analysed the region of sequence adjacent to the transposon in each isolate. This was possible for 30 isolates from the three participants and included four STs (ST80, 117, 203 and 787). The highest match in the blast database predicted that the transposon was present on the pLG1 mega-plasmid (accession numbers HM565234 and HM565172) [28] for 29/30 isolates, (100 % ID up to 5998 bp) and was adjacent to a gene encoding a cadmium efflux system accessory protein. Blast analysis of the remaining isolate (from P15) gave multiple matches to regions in *E. faecium* strain DO plasmid 2 (accession number CP003585) [29], p5753cB (GQ900487) [30], pS177 (HQ115078) [31], pF856 (JQ663598) [32] and pRUM (AF507977) [33]. To analyse the similarity between the putative pLG1 plasmid transposons, sequence reads for the 29 genomes were mapped to a reference *vanA* transposon. This revealed that the six genomes from P31 isolates shared 100 % identity with the reference, while 23 isolates from P7 were identical with the exception of the same SNP at position 8634. We also characterised variation in plasmids using *rep* typing, which demonstrated the presence of seven types (10, 11, 14, 17, 18, 20 and pMG1) with a range of 2–7 *rep* types per isolate. There was variation in the presence of *rep* types, both within and between individual STs, which extended to variation within the same individual.

### Genetic relatedness between vancomycin-resistant and -susceptible *E. faecium*

All 51 VRE-positive stools were cultured for the presence of VSE, which was identified in 25 (49 %) stools from the three participants (n = 9, 15 and 1 for P7, P15 and P31, respectively). MLST was identified from the sequence data, which assigned the isolates to nine STs. As before, P7 and P15 carried multiple STs: P7 carried ST80, ST117 and ST203 (mirroring the VRE STs in this individual) together with ST127 (a single locus variant of ST80), while P15 carried ST787 and ST18 (mirroring the VRE STs in this individual), together with two STs not represented in the VRE population (ST206 and ST17). By contrast, the VSE carried by P31 was assigned to an ST that was different from their VRE strain (ST328). *Rep* typing of the VSE isolates revealed the same *rep* types found in the VRE isolates, with a median of 6 *rep* types per isolate (range, 2–7). No *rep* types were specific to VRE or VSE. One phenotypically vancomycin susceptible isolate carried the *vanB* gene (P15-week(W)12-ST17) based on *in silico* PCR. This isolate lacked the *vanR* and *vanS* genes from whole genome sequence data and from the assembled Tn*1549* transposon. Loss of *vanR* and *vanS* from the van operon resulting in a susceptible phenotype has been described previously for *vanA*-positive isolates [32, 34]. A phylogenetic tree of the 51 VRE and 25 VSE isolates based on core genome SNPs is shown in Additional file 2. This showed that the VSE carried by P7 and P15 were closely related to their resident VRE populations demonstrating possible *in vivo* acquisition or loss of resistance, whilst as expected the VSE carried by P31 was not closely related to their VRE.

### Relatedness of *E. faecium* within a healthcare network

**Fig. 2:**
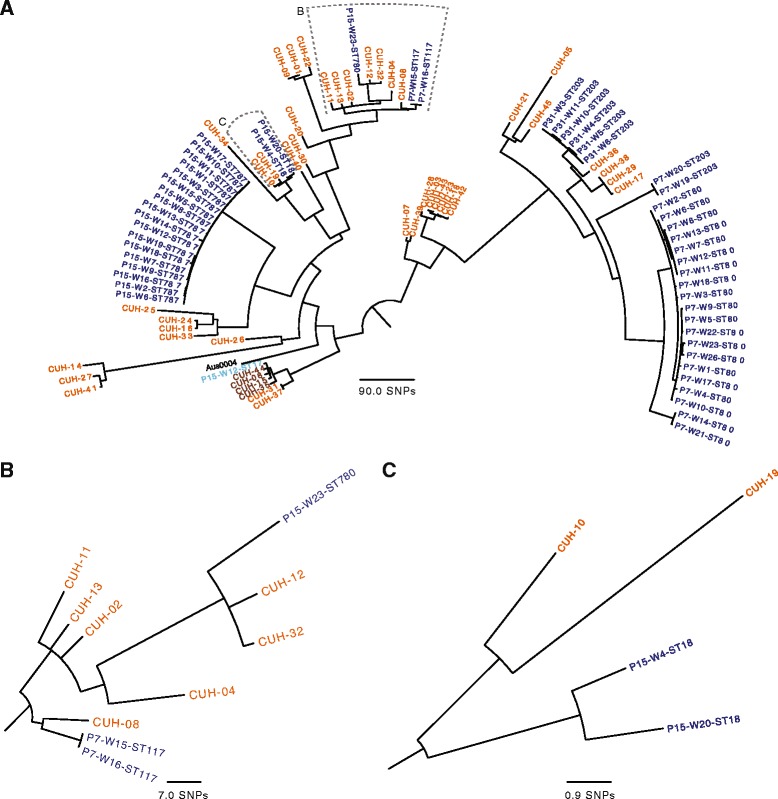
Phylogeny and relatedness *of Enterococcus faecium* isolated from networked healthcare facilities. **a** Maximum likelihood tree of 51 VRE positive isolates cultured from the stool of three study participants labelled by participant and week (dark blue), one VSE carriage isolate from the LTCF carrying *vanB* (light blue), 41 VRE isolates from patients with bloodstream infection in 2012 at the nearby acute NHS Trust (orange) and four VSE isolates carrying *vanB* from patients with bloodstream infection (brown). **b** Enlarged view of closely related isolates from P7 and P15 and isolates associated with bloodstream infection. **c** Enlarged view of tree containing closely related isolates from P15 and isolates associated with bloodstream infection

Sequence data for VRE isolates from study participants were compared with that for 45 *E. faecium* isolates from patients with bloodstream infection at the nearby acute hospital during 2012. Phenotypic susceptibility testing demonstrated that 41 isolates were vancomycin-resistant (all of which carried *vanA*), and four isolates were vancomycin-susceptible. A phylogenetic tree of the 51 VRE from stool and 45 hospital isolates based on core genome SNPs showed that carriage isolates from the three study participants were interspersed throughout the tree (Fig. 2a). There were several examples where isolates from the LTCF and hospital were highly related (Fig. 2b and c) which included two sets of isolates from P15 and one from P7. One of these clusters included two VRE isolates from P7 that were closely related to six hospital-associated VRE bloodstream isolates cultured in 2012 (minimum pairwise SNP difference of 4; range, 4–15) and one from P15 with a minimum pairwise SNP difference of 6 (Fig. 2b). P7 was not admitted to CUH during 2012, but was admitted twice during 2013 (for 1.5 and 2 months, respectively) and was also admitted to CUH on four further occasions during the study period. Comparison of transposons and sequences downstream of their insertion between the LTCF and hospital isolates was possible for five of these six hospital isolates. This revealed that the transposon sequences and downstream sequences for 4/5 were identical to the two isolates from P7. The fifth hospital isolate had two SNPs and an 89 bp insertion in the sequence downstream of the transposon. A second cluster involved VRE carried by P15 and VRE bloodstream isolates from two patients, the closest of which also differed by six SNPs (Fig. 2c). A case note review revealed that P15 was admitted to the CUH on three occasions in 2012, and had ampicillin-resistant *E. faecium* isolated from urine during the third admission. P15 was an inpatient at the same time, but not on the same ward as the two patients with bloodstream infection in this cluster (Fig. 2c). The four vancomycin-susceptible isolates were found to be carrying *vanB* by *in silico* PCR. Additionally, we noted that the single isolate positive for *vanB* isolated from P15 clustered with the four *vanB* positive hospital-associated bloodstream isolates*,* the closest of which was 12 SNPs different based on a core genome comparison. Mapping of these isolates to the Aus0004 Tn*1549* transposon demonstrated that the transposons and their downstream sequences were identical to that of the *vanB-*positive isolate from the LTCF.

## Discussion

Residents of LTCFs are at increased risk of infection from a range of multidrug-resistant organisms (MDRO) compared with the general population [35, 36]. This may be related to several factors, including frequent healthcare contact and high levels of antibiotic use. Furthermore, LTCF are often akin to a home environment, where screening for MDROs is not routinely performed and isolation of residents may be detrimental to care. The combination of these factors represents a perfect storm for the control of MDRO.

In this study, we focused on carriage and transmission of VRE, a nosocomial pathogen that has remained a persistent problem despite the raft of infection control measures introduced into UK hospitals to reduce the prevalence of MRSA and *C. difficile*. We found that 7 % of residents carried VRE in their stool on at least one occasion, a finding consistent with previous studies in Israel, Germany and the United States [6, 7, 37]. Our longitudinal study design allowed us to investigate carriage over time. This identified variability in the duration of carriage, with two long-term carriers and one individual who appeared to lose VRE carriage during the study. Intermittent and transient VRE carriage has been described previously [38, 39]. Genome sequencing was used to determine the genetic relatedness of VRE isolates. The advantage of doing so became apparent on comparing this with the results of *in silico* MLST. Reliance on MLST could have led to an incorrect link being made between two residents (P7 and P31) who both carried VRE ST203 which were not closely related on sequencing.

Sequencing of VSE in VRE-positive participants demonstrated that two individuals carried multiple VSE lineages that were closely related to their VRE isolates. This is consistent with the findings of Howden *et al*. [23] that VRE and VSE across the species are not genetically distinct. VSE strains may have gained resistance via horizontal gene transfer from colonising VRE lineages. We identified identical *vanA* transposons and insertion sites in multiple STs from P7, suggesting possible sharing of the *vanA* mobile genetic element within patients. *Rep* types were not distinct between VRE and VSE populations, and varied within STs, suggesting the presence of highly mobile plasmids.

A combination of epidemiological and sequence data allowed us to consider the source of VRE acquisition. While the LTCF environment might be expected to lead to multiple transmission events, there was no evidence for direct transmission of VRE within the study facility. Prior evidence for transmission of VRE in LTCFs is limited, with a single point prevalence study suggesting transmission between 3/188 residents based on pulse field gel electrophoresis [40]. Instead, the source of acquisition may be linked to the frequent interaction with healthcare facilities as two persistent carriers with a history of extensive healthcare contact carried multiple VRE lineages, the majority of which have been associated previously with the healthcare setting based on MLST [41]. This indicates long-term carriage (over 6 months) of hospital-associated lineages in community-based care facilities.

Comparison of WGS data for VRE from the LTCF and the acute hospital to which the participants are referred provided the opportunity to compare the relatedness of isolates drawn from different parts of the same healthcare network. This demonstrated that isolates associated with bloodstream infection were highly related to those carried by the LTCF residents. Direct transmission between the study participants and hospital patients is unlikely, but two of the participants carrying VRE were admitted to the hospital within a relevant timeframe for exposure to the circulating VRE lineages. Furthermore, repeated admission of the study participants represents a mechanism for perpetuation of these lineages. The sequence downstream of the *vanA* transposon in two of the LTCF isolates was identical to four of five hospital isolates closely related to them, providing further evidence for transmission of VRE from hospitals to community care facilities. Additionally, this region of sequence in the genetically more stable *vanB* Tn1549 transposon of the LTCF isolate matched those from the hospital also carrying *vanB*.

Our study has several limitations. First, only half of the residents in the study facility agreed to participate in the study, which may have obscured transmission events between study participants and individuals who were not recruited. Second, we only sequenced a single colony from each stool sample, which may have affected our ability to detect carriage of more than one VRE lineage. However, this risk was mitigated by repeated sampling of individual patients over time, which did demonstrate carriage of multiple lineages in long-term carriers. Evidence for concurrent carriage of multiple HCA lineages of enterococci is limited, but has implications for current screening and WGS techniques based upon single cultures. Finally, we only had access to the genome sequence of bloodstream isolates from the acute hospital, which may not be fully representative of the carriage population in this setting. Despite this, we were still able to provide evidence demonstrating that clones had been shared between the hospital and LTCF.

## Conclusions

This study has confirmed that a proportion of residents in a community-based long-term care facility carried VRE in stool, genome sequencing of which demonstrated multiple lineages in some cases but no evidence for transmission between residents. This population frequently requires admission to hospital, where they may acquire nosocomial pathogens as well as transmit existing strains to hospital patients and shed organisms into the environment. A genome comparison of VRE carriage isolates in the LTCF and VRE bloodstream isolates confirmed that VRE in the two populations were highly related. This shared population is likely to reflect two-way transmission. Tackling multidrug-resistant pathogens in hospitals requires an understanding of reservoirs and transmission of bacteria within highly connected healthcare facilities, together with infection control policies that consider this as a continuum.

## Additional files

Additional file 1:
**Table detailing the accession numbers of isolates used within the study.** (XLSX 13 kb) Additional file 2:
**Figure depicting the phylogeny and relatedness of VRE and VSE carried in stool by study participants.** (PDF 167 kb)

## References

[1] European Centre for Disease Prevention and Control. Antimicrobial resistance surveillance in Europe 2013. Annual Report of the European Antimicrobial Resistance Surveillance Network (EARS-Net). 2014. http://ecdc.europa.eu/en/publications/_layouts/forms/Publication_DispForm.aspx?List=4f55ad51-4aed-4d32-b960-af70113dbb90&ID=1205 (accessed 21st April 2015).

[2] Public Health England. Annual Epidemiological Commentary: Mandatory MRSA, MSSA and *E. coli* bacteraemia and *C. difficile* infection data 2013/14. 2014. https://www.gov.uk/government/statistics/mrsa-mssa-and-e-coli-bacteraemia-and-c-difficile-infection-annual-epidemiological-commentary (accessed 10th April 2015).

[3] Public Health England. Voluntary surveillance of *Enterococcus* spp. bacteraemia, England, Wales and Northern Ireland: 2014. 2014. https://www.gov.uk/government/publications/enterococcus-spp-bacteraemia-voluntary-surveillance (accessed 7th April 2015).

[4] Cheah ALY, Spelman T, Liew D, Peel T, Howden BP, Spelman D (2013). Enterococcal bacteraemia: factors influencing mortality, length of stay and costs of hospitalization. Clin Microbiol Infect.

[5] Diaz Granados CA, Zimmer S, Klein M, Jernigan JA (2005). Comparison of mortality associated with vancomycin-resistant and vancomycin-susceptible enterococcal bloodstream infections: A meta-analysis. Clin Infect Dis..

[6] Benenson S, Cohen MJ, Block C, Stern S, Weiss Y, Moses AE (2009). Vancomycin-resistant Enterococci in long - term care facilities. Infect Control Hosp Epidemiol.

[7] Gruber I, Heudorf U, Werner G, Pfeifer Y, Imirzalioglu C, Ackermann H (2013). Multidrug-resistant bacteria in geriatric clinics, nursing homes, and ambulant care--prevalence and risk factors. Int J Med Microbiol.

[8] Kahvecioglu D, Ramiah K, McMaughan D, Garfinkel S, McSorley VE, Nguyen QN (2014). Multidrug-resistant organism infections in US nursing homes: a national study of prevalence, onset, and transmission across care settings, October 1, 2010-December 31, 2011. Infect Control Hosp Epidemiol..

[9] Reuter S, Ellington MJ, Cartwright EJP, Köser CU, Török ME, Gouliouris T (2013). Rapid bacterial whole-genome sequencing to enhance diagnostic and public health microbiology. JAMA Intern Med.

[10] Hall BG, Ehrlich GD, Hu FZ (2010). Pan-genome analysis provides much higher strain typing resolution than multi-locus sequence typing. Microbiology.

[11] Snitkin ES, Zelazny AM, Thomas PJ, Stock F, Henderson DK, NISC Comparative Sequencing Program (2012). Tracking a hospital outbreak of carbapenem-resistant *Klebsiella pneumoniae* with whole-genome sequencing. Sci Transl Med.

[12] Harris SR, Feil EJ, Holden MTG, Quail MA, Nicker EK, Chantratita N (2010). Evolution of MRSA during hospital transmission and intercontinental spread. Science.

[13] Török ME, Harris SR, Cartwright EJ, Raven KE, Brown NM, Allison ME (2014). Zero tolerance for healthcare-associated MRSA bacteraemia: is it realistic?. J Antimicrob Chemother.

[14] Harris SR, Cartwright EJP, Török ME, Holden MTG, Brown NM, Ogilvy-Stuart AL (2013). Whole-genome sequencing for analysis of an outbreak of meticillin-resistant Staphylococcus aureus: a descriptive study. Lancet Infect Dis.

[15] Golubchik T, Batty EM, Miller RR, Farr H, Young BC, Larner-Svensson H (2013). Within-host evolution of *Staphylococcus aureus* during asymptomatic carriage. PLoS One.

[16] Stoesser N, Sheppard AE, Moore CE, Golubchik T, Parry CM, Nget P (2015). Extensive within-host diversity in fecally carried extended-spectrum beta-lactamase-producing *Escherichia coli*: implications for transmission analyses. J Clin Microbiol.

[17] SMALT: Pairwise Sequence Alignment Program. Wellcome Trust Sanger Institute, Hinxton, UK http://www.sanger.ac.uk/science/tools/smalt-0 (accessed 1st June 2015).

[18] Croucher NJ, Page AJ, Connor TR, Delaney AJ, Keane JA, Bentley SD (2015). Rapid phylogenetic analysis of large samples of recombinant bacterial whole genome sequences using Gubbins. Nucleic Acids Res.

[19] Stamatakis A (2006). RAxML-VI-HPC: maximum likelihood-based phylogenetic analyses with thousands of taxa and mixed models. Bioinformatics.

[20] Multi Locus Sequence Typing: *Enterococcus faecium.* Imperial College, London http://efaecium.mlst.net/ (accessed 13th May 2015).

[21] Dutka-Malen S, Evers S, Courvalin P (1995). Detection of glycopeptide resistance genotypes and identification to the species level of clinically relevant Enterococci by PCR. J Clin Microbiol.

[22] Depardieu F, Perichon B, Courvalin P (2004). Detection of the van alphabet and identification of enterococci and staphylococci at the species level by multiplex PCR. J Clin Microbiol.

[23] Howden BP, Holt KE, Lam MM, Seemann T, Ballard S, Coombs GW, et al. Genomic insights to control the emergence of vancomycin-resistant enterococci. MBio. 2013;4(4). doi:10.1128/mBio.00412-13. 10.1128/mBio.00412-13 PMC3747580 23943759

[24] Rutherford K, Parkhill J, Crook J, Horsnell T, Rice P, Rajandream MA (2000). Artemis: sequence visualization and annotation. Bioinformatics.

[25] Lam MMC, Seeman T, Tobias NJ, Chen H, Haring V, Moore RJ (2013). Comparative analysis of the complete genome of an epidemic hospital sequence type 203 clone of vancomycin-resistant *Enterococcus faecium*. BMC Genomics..

[26] Jensen LB, Garcia-Migura L, Valenzuela AJS, Løhr M, Hasman H, Aerestrup FM (2010). A classification system for plasmids from enterococci and other Gram-positive bacteria. J Microbiol Methods.

[27] Wardal E, Gawryszewka I, Hryniewicz W, Sadowy E (2013). Abundance and diversity of plasmid-associated genes among clinical isolates of *Enterococcus faecalis*. Plasmid.

[28] Laverde Gomez JA, van Schaik W, Freitas A, Coque T, Weaver KE, Francia MV (2011). A multiresistance megaplasmid pLG1 bearing *hyl*_*Efm*_ genomic island in hospital *Enterococcus faecium* isolates. Int J Med Microbiol.

[29] Qin X, Galloway-Peña JR, Sillanpaa J, Roh JH, Nallapareddy SR, Chowdhury S (2012). Complete genome sequence of *Enterococcus faecium* strain TX16 and comparative genomic analysis of *Enterococcus faecium* genomes. BMC Microbiol..

[30] Li X, Alvarez V, Harper WJ, Wang HH (2011). Identification of a persistent, TA-independent tetracycline resistance-encoding plasmid from a dairy *Enterococcus faecium* isolate. Appl Environ Microbiol.

[31] Halvorsen EM, Williams JJ, Bhimani AJ, Billings EA, Hergenrother PJ (2011). Txe, an endoribonuclease of the enterococcal Axe-Txa toxin-antitoxin system, cleaves mRNA and inhibits protein synthesis. Microbiology.

[32] Szakacs TA, Kalan L, McConnell MJ, Eshaghi A, Shahinas D, McGeer A (2014). Outbreak of vancomycin-susceptible *Enterococcus faecium* containing the wild-type *vanA* gene. J Clin Microbiol.

[33] Grady R, Hayes F (2003). Axe-Txe, a broad-spectrum proteic toxin-antitoxin system specified by a multidrug-resistant, clinical isolate of *Enterococcus faecium*. Mol Microbiol.

[34] Coburn B, Low DE, Patel SN, Poutanen SM, Shahinas D, Eshaghi A (2014). Vancomycin-variable *Enterococcus faecium: In vivo* emergence of vancomycin resistance in a vancomycin-susceptible isolate. J Clin Microbiol.

[35] Lucet JC, Grenet K, Armand-Lefevre L, Harnal M, Bouvet E, Regnier B (2005). High prevalence of carriage of methicillin-resistant *Staphylococcus aureus* at hospital admission in elderly patients: implications for infection control strategies. Infect Control Hosp Epidemiol.

[36] Pop-Vicas AE, D'Agata EM (2005). The rising influx of multidrug-resistant Gram-negative bacilli into a tertiary care hospital. Clin Infect Dis..

[37] Pop-Vicas AE, Mitchell SL, Kandel R, Schreiber R, D'Agata EM (2008). Multidrug-resistant gram-negative bacteria in a long-term care facility: prevalence and risk factors. J Am Geriatr Soc.

[38] Roghmann M-C, Qaiyumi S, Schwalbe R, Morris JG (1997). Natural history of colonization with vancomycin-resistant *Enterococcus faecium*. Infect Control Hosp Epidemiol.

[39] Montecalvo MA, Jarvis WR, Uman J, Shay DK, Petrullo C, Horowitz HW (2001). Natural history of colonization with vancomycin-resistant *Enterococcus faecium*. Infect Control Hosp Epidemiol.

[40] Wendt C, Krause C, Xander LU, Loffler D, Floss H (1999). Prevalence of colonization with vancomycin-resistant enterococci in various population groups in Berlin, Germany. Infect Control Hosp Epidemiol..

[41] Willems RJL, van Schaik W (2009). Transition of *Enterococcus faecium* from commensal organism to nosocomial pathogen. Future Microbiol.

