# Supplementary figures and images for: Whole-genome sequencing reveals transmission of vancomycin-resistant Enterococcus faecium in a healthcare network
Source: Genome Med. 2016 Jan 12;8:4. doi: 10.1186/s13073-015-0259-7 (PMC4709893; doi:10.1186/s13073-015-0259-7)

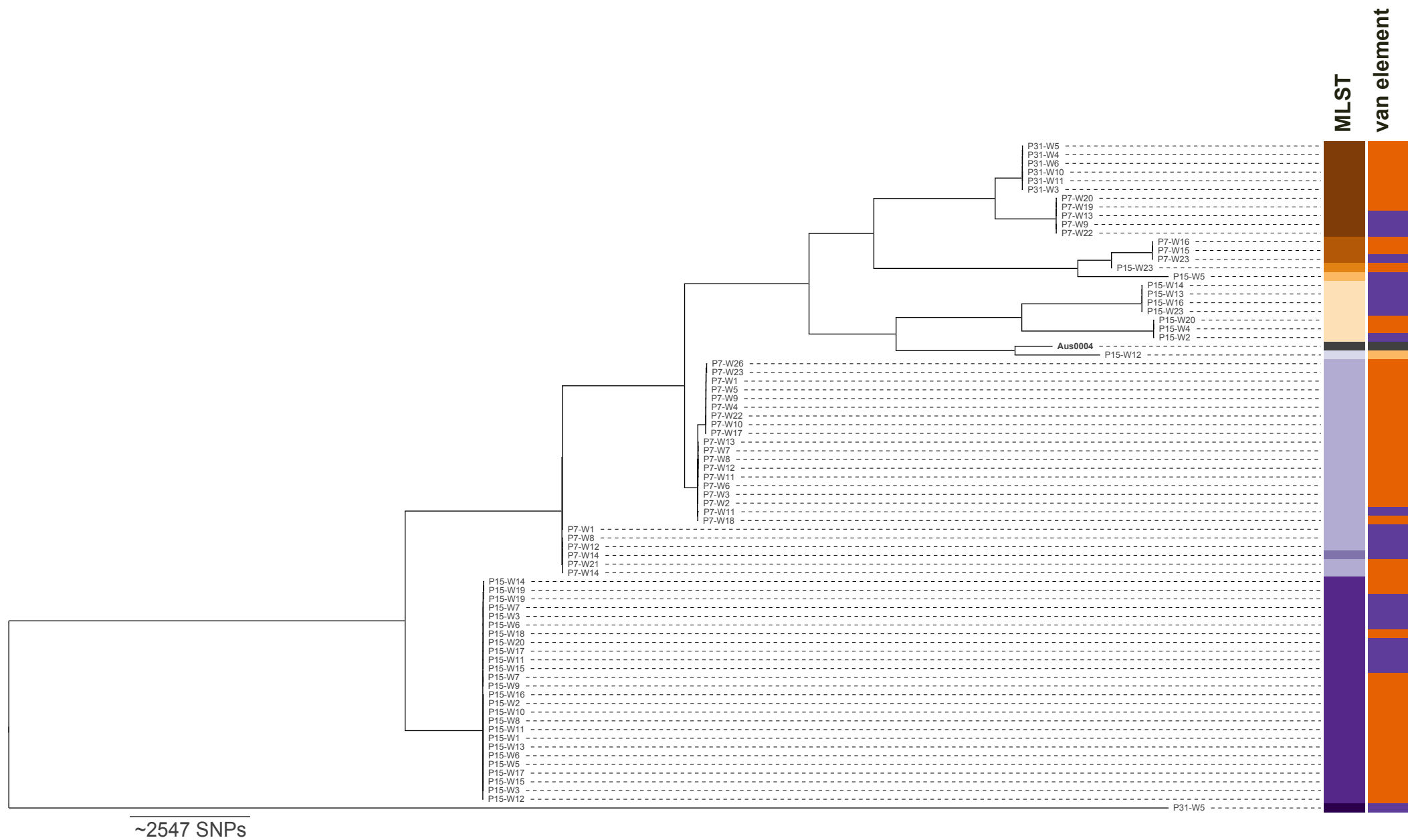

**Key:** MLST: 17 18 80 117 127 203 206 328 780 787  
 van element: VSE vanA vanB

Supplement: Additional file 2: — Figure depicting the phylogeny and relatedness of VRE and VSE carried in stool by study participants. (PDF 167 kb) [file 13073_2015_259_MOESM2_ESM.pdf]
